# Controlling the Formation of Polyelectrolyte Complex Nanoparticles Using Programmable pH Reactions

**DOI:** 10.1021/acs.macromol.2c01431

**Published:** 2022-12-16

**Authors:** Christian C. M. Sproncken, Berta Gumí-Audenis, Sanam Foroutanparsa, José Rodrigo Magana, Ilja K. Voets

**Affiliations:** Laboratory of Self-Organizing Soft Matter, Department of Chemical Engineering and Chemistry, Institute for Complex Molecular Systems, Eindhoven University of Technology, P.O. Box 513, 5600 MBEindhoven, The Netherlands

## Abstract

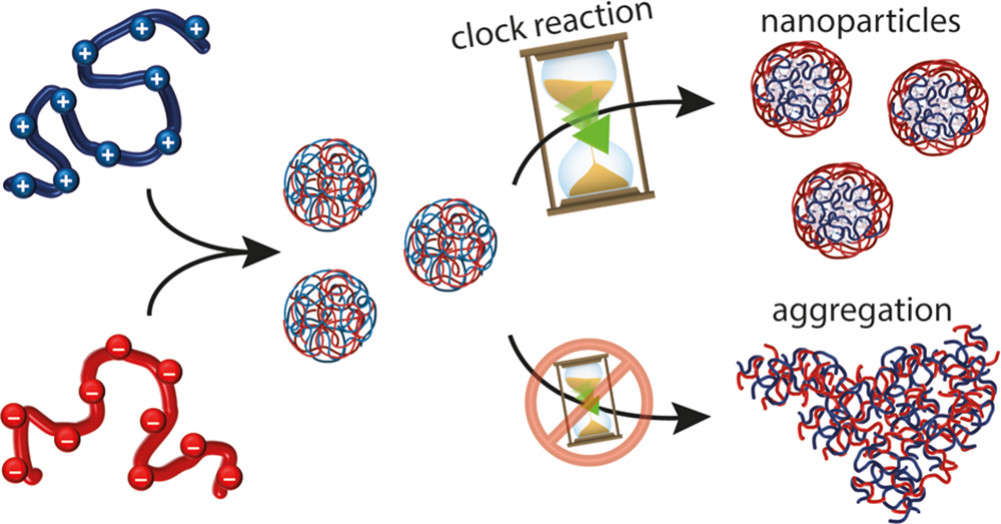

Enabling complexation of weak polyelectrolytes, in the presence of a programmable pH-modulation, offers a means to achieve temporal control over polyelectrolyte coassembly. Here, by mixing oppositely charged poly(allylamine hydrochloride) and poly(sodium methacrylate) in a (bi)sulfite buffer, nanoscopic complex coacervates are formed. Addition of formaldehyde initiates the formaldehyde-sulfite clock reaction, affecting the polyelectrolyte assembly in two ways. First, the abrupt pH increase from the reaction changes the charge density of the polyelectrolytes and thus the ratio of cationic and anionic species. Simultaneously, reactions between the polyamine and formaldehyde lead to chemical modifications on the polymer. Interestingly, core–shell polymeric nanoparticles are produced, which remain colloidally stable for months. Contrastingly, in the same system, in the absence of the clock reaction, aggregation and phase separation occur within minutes to days after mixing. Introducing an acid-producing reaction enables further temporal control over the coassembly, generating transient nanoparticles with nanoscopic dimensions and an adjustable lifetime of tens of minutes.

## Introduction

Complex coacervation occurs when a mixture of charged (macro)molecules separates into two different liquid phases: a dilute phase depleted from most of the macromolecules and a dense phase enriched in both polyelectrolytes. Nature exploits coacervation for (transient) compartmentalization of compounds into nonmembrane-bound subcellular structures.^1,2^ Inspired by this evolutive strategy, synthetic systems are designed to mimic organelles^3^ or protocells.^4−6^ Furthermore, coacervation can also be used to replicate food microstructure^7−9^ and encapsulate active cargo.^8,10−12^

When coacervation commences, the initially formed complex nanodroplets grow, minimizing the system’s total surface energy until they separate into two macroscopic phases. Controlling the size of complex coacervate droplet phases is generally achieved by adding a polymeric stabilizer. For example, covalently anchoring a water-soluble, neutral block onto (at least) one of the polyelectrolytes results in the formation of nanometric-sized micelles, so-called complex coacervate core micelles. The particle size can be tuned by, e.g., varying the block lengths and ratios between the charged and neutral parts. For stabilization of larger complex coacervate droplets with diameters of tens of micrometers, polymers or lipids can be added once the desired size is reached. These attach onto the coacervate surface, blocking further growth of the particles.^6,13−15^

Size control of polyion particles can also be achieved through kinetic arrest. Kinetic traps can be purposely introduced in the coassembly pathway in a number of ways: i.e., through assembly at low ionic strength^16^ and/or via strong polyelectrolytes with a fixed high charge density^17^ or hydrophobic polyelectrolytes^18^ which become water-insoluble upon neutralization. Alternatively, the pathways of particle formation can be tuned. Based on the latter, we recently achieved the formation of colloidally stable cross-linked nanoparticles without the use of steric stabilizers by using pH-modulated clock reactions.^19^ There, poly(allylamine hydrochloride)/sulfite complexes underwent a sudden pH change caused by the well-known formaldehyde-sulfite (F-S) clock reaction.^20−22^ The coacervate-like complexes act as a supramolecular template, bringing the amine groups closer together, thereby increasing the local density of reactive sites, which are covalently cross-linked by their subsequent reaction with formaldehyde at basic pH. Encouraged by these results, we explored whether pH-modulated clock reactions can be used as an alternative strategy to control the growth of polyion coacervates and create electrostatically assembled colloidally stable objects with nano- and microscopic dimensions. Here, we drive the electrostatic coassembly of two pH-responsive polyelectrolytes, poly(allylamine hydrochloride) (PAH) and poly(sodium methacrylate) (PSM), using the F-S clock reaction. Nanoscopic complex coacervates form by mixing these polymers, which, in the absence of the clock reaction, macroscopically phase-separate or aggregate within minutes to days. We show that the reaction network can be utilized to restrain the growth of the coacervate and to preserve its nanoscopic size, resulting in polymeric nanoparticles that neither grow nor shrink over months. Additionally, this strategy is extended to control not only the dimensions but also the lifetime of the nanoparticles using a combination of F-S clock and acid-producing hydrolysis of 1,3-propanesultone.

## Experimental Section

### Materials and Sample Preparation

Polyallylamine hydrochloride (PAH, *M*_w_ ≈ 120,000–200,000 g mol^–1^) was purchased from Alfa Aesar (USA). Formaldehyde (ACS reagent, 37 wt % in H_2_O), poly(sodium methacrylate) (PSM, *M*_w_ = 4000–6000 g mol^–1^, 40 wt % in H_2_O), sodium chloride (99+%), and 1,3-propanesultone (PrS, 98%) were purchased from Sigma-Aldrich (USA). Sodium sulfite (98.5%), sodium bisulfite (mixture of NaHSO_3_ and Na_2_S_2_O_5_), and sodium nitrate (98.5%) were obtained from Acros Organics (USA) while sodium hydroxide (pellets Emprove) were acquired from Merck (Germany). Stock solutions of each reagent were prepared in ultrapure water (18.2 MΩ cm, Arium water purification system, Sartorius, Germany), which was bubbled with dry nitrogen gas for 15 min before use. Samples were prepared by mixing these solutions in the desired ratios, obtaining a total volume of 3.0 mL. Since electrostatic coassembly involves two species that are mixed, it is common to express the concentration as total concentration of chargeable monomers:
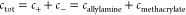
1Here, in all experiments where the polymers are mixed, the concentrations are specified as *c*_+/–_, indicating the molar concentration of chargeable cations or anions. We always mix these oppositely charged polymers in a 1:1 ratio, so that total chargeable monomer concentration is therefore two times this concentration:
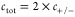
2

To ensure reproducibility, sodium (bi)sulfite solutions were always used within 2 h after preparation, after which slow oxidation would become apparent. When PrS was used, the compound was melted in a 40 °C water bath due to its melting point of 31 °C, after which the desired amount of liquid was added to the samples prior to formaldehyde addition. All experiments were performed at 21.0 ± 1.0 °C unless specifically stated otherwise.

### pH Measurements

The pH measurements were performed in a 3.0 mL sample volume while mildly stirring at 100 rpm, and using a SevenCompact S220 pH meter, equipped with an Inlab Micro electrode (Mettler Toledo, USA). Easydirect pH software was used to export the pH value every 2 s.

### Zeta Potential Measurements

Electrophoretic mobility was measured using Omega Z cuvettes in a Litesizer 500 (Anton Paar GmbH, Austria). The same 3.0 mL samples were prepared, and 300 μL was taken out to determine zeta potentials “before the clock”. After the addition of formaldehyde and stirring for 3 min, a new aliquot of 300 μL was taken to measure the zeta potential “after the clock”. The voltage applied was 86.2 ± 0.8 mV and a Smoluchowski approximation was used to calculate the zeta potential from the electrophoretic mobility.

### Turbidity Measurements

Transmission values were recorded using a time-dependent measurement on a V-650 UV–VIS spectrophotometer equipped with Spectra Manager software (Jasco GmbH, Germany). The acquisition was performed using 10 mm polystyrene cuvettes at a wavelength of 532 nm with a collection interval of 2 s while stirring at 100 rpm. The turbidity was then calculated from the measured transmission (*T* in %) since it presents a more convenient measure of the complex coacervation using
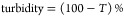
3

### Light Scattering Measurements and Analysis

Light scattering experiments were performed on an ALV/CGS-3 MD-4 Goniometer System (ALV GmbH, Germany), equipped with a 50 mW Nd:YAG laser operating at 532 nm. The temperature was regulated at 20.0 ± 0.2 °C using a Lauda RM6-S Refrigerated Circulating Bath. The light scattering intensity was recorded at 90°. Characteristic decay rates (Γ) obtained from the normalized intensity autocorrelation function were used to calculate the translational diffusion coefficient (*D*_T_). The distributions of the particles’ hydrodynamic radii (*R*_H_) were determined using the CONTIN method and the Stokes–Einstein relation. For in situ measurement of pH and light scattering, a specifically designed glass cell was used, in which 12.0 mL of the sample can be stirred by an overhead stirring motor, while inserting the pH electrode under an angle from an entry point at the side. Constant stirring ensured the full mixing of the compounds directly after addition without interfering with the measurements.

### Transmission Electron Microscopy

Images were acquired using a FEI Tecnai 20, type Sphera TEM instrument operating at 200 kV (LaB6 filament) with a bottom-mounted 1024 × 1024 Gatan msc 794 CCD camera. Briefly, samples were prepared by first incubating the coacervates for 5 min on top of glow-discharged carbon-coated copper 200-mesh TEM grids (CF200-Cu, Aurion). Excess liquid was carefully wicked away from beneath the TEM grids. Samples were then subsequently negatively stained by applying 10 μL of uranyl acetate solution (filtered, 2% w/v) on top and incubating for 90 s. Finally, excess liquid was wicked away from the edge of the TEM grids and the specimens were allowed to air-dry for at least 3 h with light ventilation. The TEM micrographs were analyzed using ImageJ. The outlines of the particles were manually drawn for 105 different particles in 15 images taken at different magnifications, and the areas within the outlines were extracted. From these areas, the equivalent radius of a circle with the same area was calculated and these values were reported as the sizes of the particles.

## Results

Aiming to elucidate whether pH-regulating reaction networks can be employed to prepare coacervates with tailored dimensions and high stability, we selected two common polyelectrolytes, poly(allylamine hydrochloride) (PAH) and poly(sodium methacrylate) (PSM), for complexation. Since both are weak polyelectrolytes, their charge densities depend on the pH of the solution. The polymers were mixed at a total polymer concentration, *c*_tot_, between 2 and 16 × 10^–3^m and a polyelectrolyte mixing ratio close to unity (*c*_+_ = *c*_–_) considering all chargeable monomers.

The formaldehyde-sulfite (F-S) clock reaction was used to program a well-defined and sudden change in pH. Briefly, the F-S reaction consists of a sulfite/bisulfite buffer at a pH between 5 and 6, of which the sulfite ions react quickly with formaldehyde, producing hydroxide. The OH^–^ ions are rapidly scavenged by bisulfite, generating in turn more sulfite ions. The change in pH in the first phase of the reaction network is therefore gradual, until bisulfite is completely consumed. At this point, the net production of hydroxide increases the pH to values between 10 and 11. This delay time before the sudden pH rise can be controlled by varying formaldehyde concentration, which we set to 0.1 M to obtain a *t*_lag_ of about 50 s. In our experiments, PSM was first added to a solution containing 1:10 sodium sulfite to sodium bisulfite (0.005/0.05 m), followed by PAH and finally formaldehyde. Initially, at pH = 5.7, positively charged polymers are present in excess as less than 50% of the methacrylate monomers are charged (pH < pKa), while the degree of ionization (α) of PAH is over 90% (Figure S1).^23,24^ Conversely, negatively charged species are most abundant after the completion of the clock reaction, where α_+_ < 25%, whereas the PSM has accumulated more charge: α_–_ > 99%.

To study the degree of complexation during the stages of the clock reaction, we assessed the turbidity of the samples over time. The instantaneous clouding of the system upon adding PAH at the beginning of the reaction before the addition of formaldehyde indicates that complexation already occurs at pH = 5.7.

**Figure 1:**
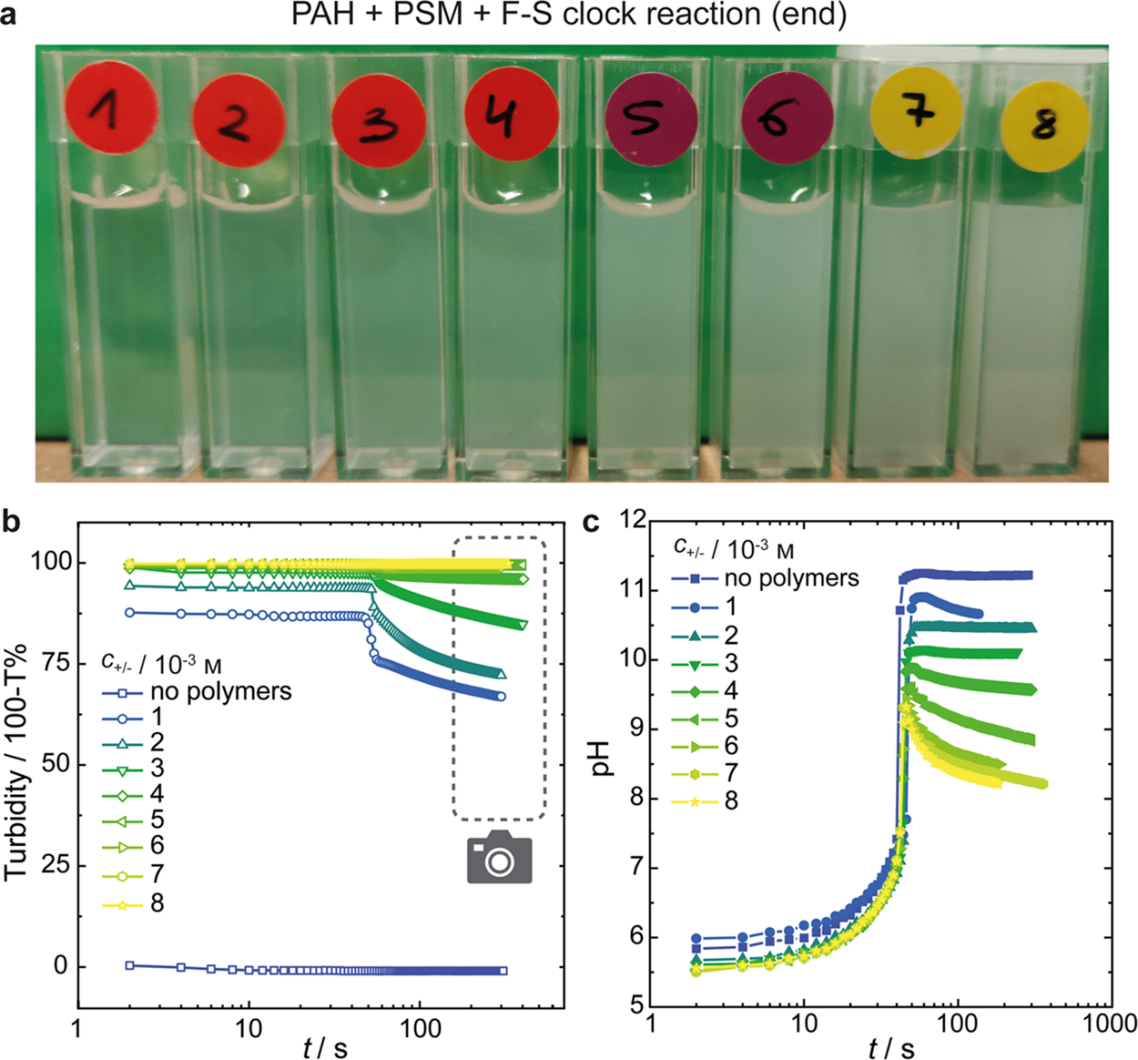
Effects of mixing PSM and PAH, at concentrations up to 8 × 10^–3^m each in stoichiometric charge ratio, with the components of the F-S clock reaction: (a) photographs of the samples taken after completion of the clock reaction, as indicated by the region within the dashed line in panel b. Measurements of the changes in (b) turbidity and (c) pH, starting from the addition of formaldehyde.

As expected, the final turbidity (when the reaction is completed) increases with the polymer concentration (Figure 1a). A closer look reveals a significant decrease in turbidity around *t* = 52 s for chargeable monomer concentrations up to 3 × 10^–3^m, which coincides with *t*_lag_ for the sharp rise in pH (Figure 1b). This is attributed to complex coacervate dissociation due to the reduction of the net positive charge of PAH at these pH values. For the highest polymer concentrations used here, such a transition cannot be observed, because 0% of the light is transmitted through the sample both before and after the clock reaction. The total polymer concentration also impacts the pH profiles (Figure 1c). For virtually all concentrations, we detect a sharp increase in pH, after which it passes through a maximum and finally drops to a nearly constant value, ranging from 0.2 to 2.1 pH units below the maximum value. Both the maximum and plateau values, at t_end_, depend on the polyelectrolyte concentration. Thus, the pH window generated by the F-S clock is strongly dampened at high polyelectrolyte concentrations, due to the release of protons from the polyelectrolytes.

**Figure 2:**
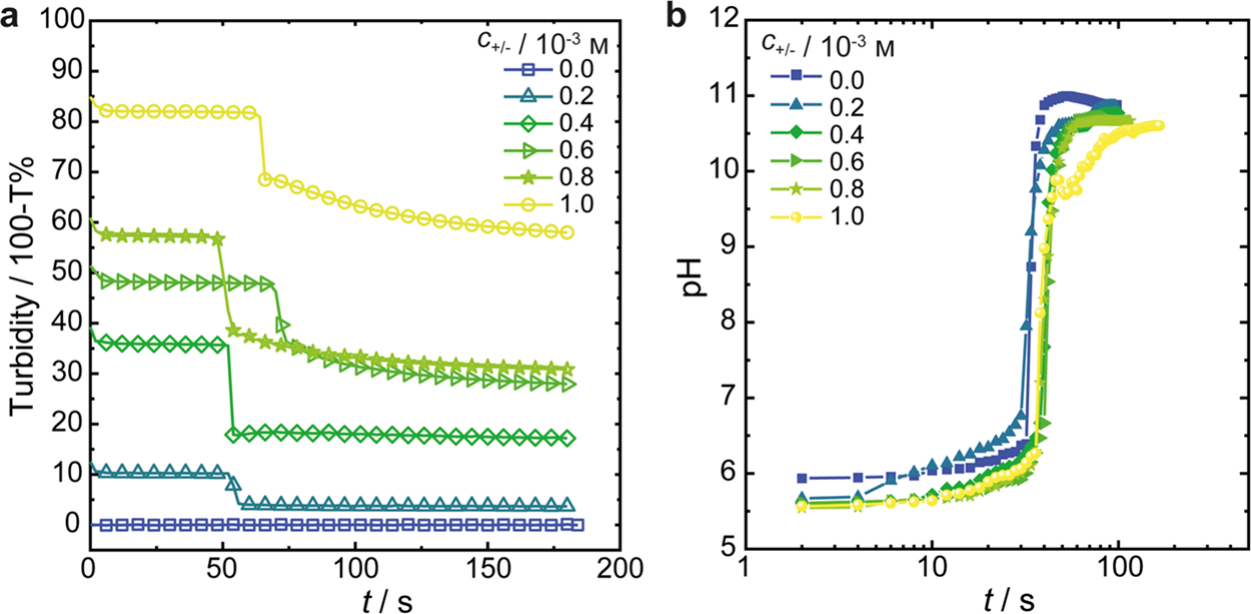
Effects of low concentrations, up to 10^–3^m, of both PAH and PSM on the (a) pH and (b) turbidity of the solutions during the course of the clock reaction. Temporal evolution of pH is only very slightly affected by polymer concentration while the turbidity increases with increasing concentration, and for each sample, a drop in turbidity occurs after the lag time of the clock reaction.

To minimize interference by the polyelectrolytes on pH-regulation by the F-S clock and to facilitate monitoring by turbidimetry, we repeated these experiments at lower polymer concentrations. The difference in turbidity is visible to the naked eye when working at polymer concentrations up to *c*_+/–_ = 10^–3^m, (Figure 2a) and pH profiles that no longer show a peak but are instead reminiscent of the profile in the absence of both polyelectrolytes (Figure 2b). At the end of the reaction, samples displayed a bluish iridescence indicative of Tyndall scattering from particles sizes in the nanometer range. Again, the turbidity is higher at the higher polymer content, indicative of a higher amount of particles and/or particles that are larger in size.

**Figure 3:**
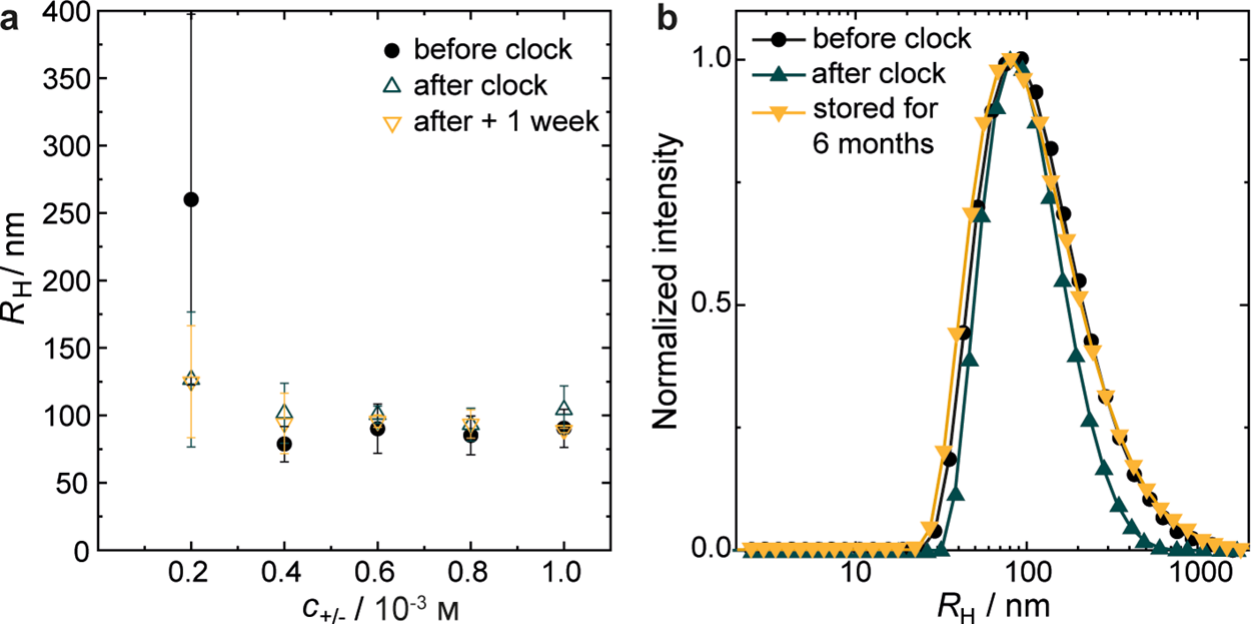
Dynamic light scattering analyses: (a) hydrodynamic radii (*R*_H_) of polyelectrolyte complexes before and after the F-S clock reaction and after storing the samples for 1 week. Error bars indicate standard deviations calculated from at least three duplicate samples. (b) Comparison of normalized size distributions before addition of formaldehyde in the (bi)sulfite buffer, after completion of the clock reaction and after 6 months of storage at ambient conditions, for samples containing 10^–3^m of both PAH and PSM.

Multi-angle dynamic light scattering (DLS) measurements were performed to determine the hydrodynamic radii (*R*_H_) of the complexes before and after the clock reaction. We study low polymer concentrations (*c*_+/–_ ≤ 10^–3^m) to ensure that the samples are sufficiently transparent, so that multiple scattering is negligible (Figure 3). One comparison, to show the negligible effect of increased concentration, at least in this 10^–3^m range, is shown for a sample containing 8 × 10^–3^m, in Figure S2. Before the clock reaction, the dimensions of the complexes are essentially the same, regardless of concentration (Figure 3a) with a *R*_H_ = 90 ± 14 nm. However, these samples are not stable in such buffers without formaldehyde for an extended period of time (*t* > 2 h). Bisulfite oxidizes to (hydrogen) sulfate, which acidifies the solution (pH < 3), causing dissolution of the polyelectrolyte complexes as α_–_ < 0.01. Preparation of complexes with the same PSM/PAH concentrations at a pH of 5.7 and similar ionic strength (*I* = 0.06 m) in sodium chloride or sodium nitrate solutions fails to produce colloidally stable particles and instead results in aggregation and precipitation of the polymer complexes after only a few days (Figure S3). Interestingly, we record very similar mean values and size distributions (Figure 3b) directly after (*R*_H_ = 104 ± 18 nm) and one week after (*R*_H_ = 89 ± 2 nm) the clock reaction, when stored at the alkaline conditions set by the F-S clock. Clearly, the particle sizes are virtually independent of the polymer concentration and are fairly constant over time. In fact, the size distributions of the system at 10^–3^m are essentially congruent even after 6 months of storage (Figure 3b). This unusually high colloidal stability contrasts sharply with the tendency of the nascent polyelectrolyte complexes to aggregate or coalesce, which results in an increase in the mean particle size over time. It is worth noting that complexes prepared by direct mixing of polyelectrolyte solutions in (bi)sulfite buffer, followed by the addition of concentrated NaOH to raise the pH to 10.5 also result in an unstable system. DLS reveals that their size (distribution) is not constant during storage. Instead, the particle size significantly increases upon the addition of base (Figure S3) and large aggregates, visible to the naked eye, were observed within a few days after preparation. With manual addition of the concentrated base, the jump in pH is similarly sudden as when employing the clock reaction, but the main difference lies in the homogeneity of the pH increase. During the clock reaction, hydroxide is produced everywhere in the system at a similar rate, while adding concentrated NaOH by pipetting makes the change in pH less homogeneous. This demonstration tells us that the observed phenomena cannot be explained solely by an increase in pH and that the clock reaction provides a specific set of conditions that lead to these colloidally stable nanoparticle suspensions. We suggest that the sudden change in pH alters the coacervate nanostructure, in a way that suppresses aggregation, coalescence, and macrophase separation. Additionally, it is likely that formaldehyde mediated cross-linking of PAH, as demonstrated previously,^19^ contributes to the enhanced stability so that particle sizes are preserved for at least 6 months.

**Figure 4:**
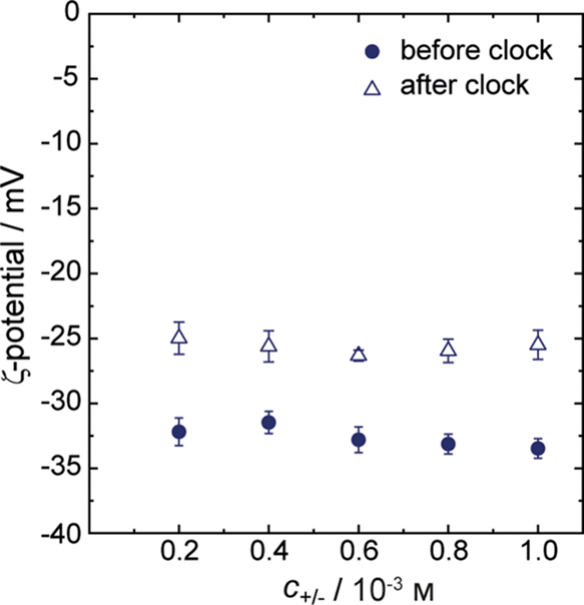
Zeta potentials of PAH/PSM complex particles measured before and after the F-S clock reaction; error bars indicate standard deviations calculated from three duplicate samples.

To shed light on the origin of the high colloidal stability and ascertain whether it is related to excess surface charge, acquired during the clock reaction, we measured the electrophoretic mobility of the complex coacervates and used these values to determine their zeta potentials. Markedly negative zeta potentials were found for all particles, irrespective of polymer concentration, both before and after the clock reaction (Figure 4). The negative values (ca. −33 mV) before the clock reaction are unexpected. The polymer mixing ratio relative to the total amount of chargeable monomer is 1:1, while their relative charge densities determine the actual charge ratio. At the initial pH = 5.7, α ∼ 95% for PAH and α ∼ 40% for PSM; hence, electrophoretic mobility measurements should output a positive zeta potential value. Tentatively, we attribute the negative charge of the complexes before the reaction to co-complexation (of PAH) with sulfite ions. Indeed, PAH can interact with sulfite ions due to their multivalent character to form aggregates.^19^ The negative zeta potentials observed after completion of the clock reaction (ca. −25 mV) are in line with expectation. The charge balance is shifted as the pH increase to 10.5 results in *c*_+_α_+_ = 0.22 × 10^–3^m and *c*_–_α_–_ = 0.99 × 10^–3^m. The excess of negatively charged monomers is increased further due to reactions of the primary amines of PAH since the chemically modified monomers no longer carry a charge. Note that all (multivalent) sulfite ions are consumed during the reaction, so these no longer contribute to the negative surface charge of the complexes at *t*_end_.

**Figure 5:**
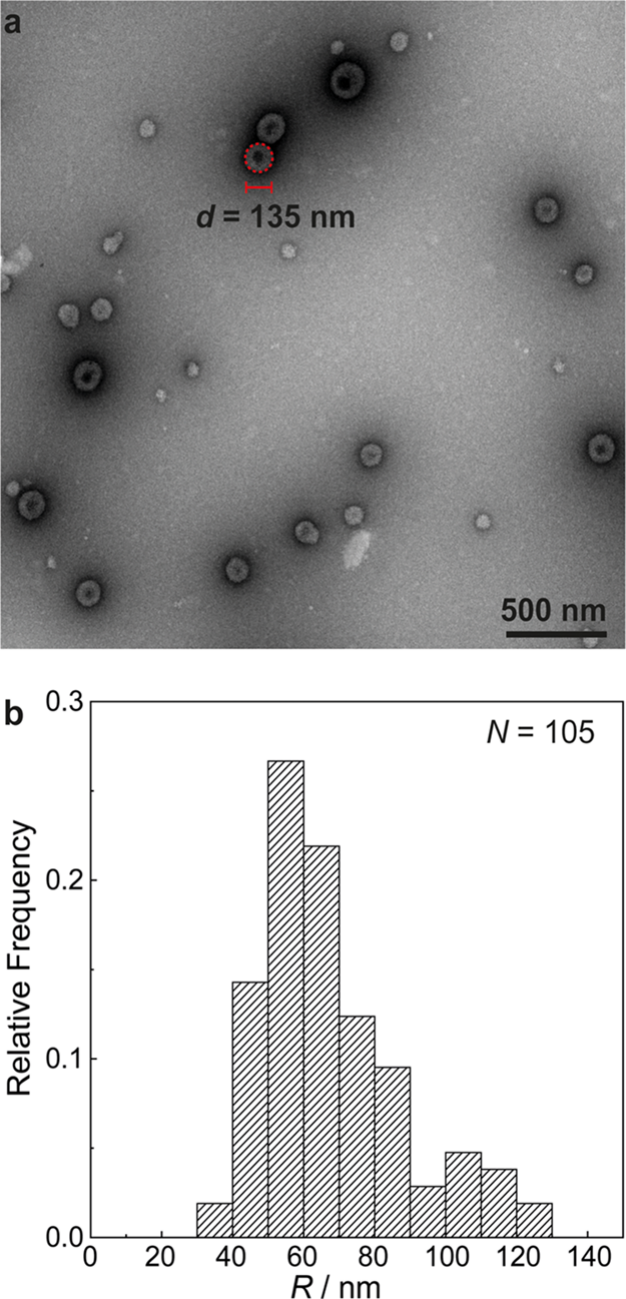
(a) Transmission electron micrographs of samples taken after the clock reaction, containing *c*_+/–_ = 10^–3^m of both PAH and PSM. Scale bar represents 500 nm. Red circle indicates an example of manual size determination for one particle (details in the Experimental Section); (b) distribution of nanoparticle radii obtained from TEM analysis. Total of 105 data points were collected from 15 images with various magnifications.

To have a better insight in the particles structure, we used transmission electron microscopy (TEM), which revealed spherical particles with a core-shell architecture (Figure 5a). Manual sizing on the order of 100 particles yields radii ranging from 38 to 127 nm and a mean radius of 67 ± 20 nm (Figure 5b). We assign these significantly smaller sizes obtained by TEM, compared to the *R*_H_ obtained by DLS (ca. 100 nm), to the dehydration of the nanoparticles during TEM sample preparation.

Based on the results from DLS, zeta potential, and TEM, we speculate that complex coacervation of PAH and PSM, manipulated by the F-S clock, generates core–shell particles with PSM chains decorating the particle surface. As the reaction is driven toward completion, PAH reacts with formaldehyde, as previously reported,^19^ and becomes less charged by the pH increase. As a consequence, some PSM chains are expelled to restore local charge neutrality. Those PSM chains that remain partially bound, by electrostatic interactions and/or entanglements, may redistribute over the particle surface to result in a negatively charged outer layer. Consequently, the particles would acquire a sufficiently dense and charged polymer shell to act as an efficient electrostatic and steric barrier against aggregation. The repulsive interactions between the like-charged PSM chains could lead to stretching of those chains, explaining why the hydrodynamic size of the particles is not reduced, compared to that of the coacervates before the clock reaction.

### Transient Programming of Coassembly and Disassembly

**Figure 6:**
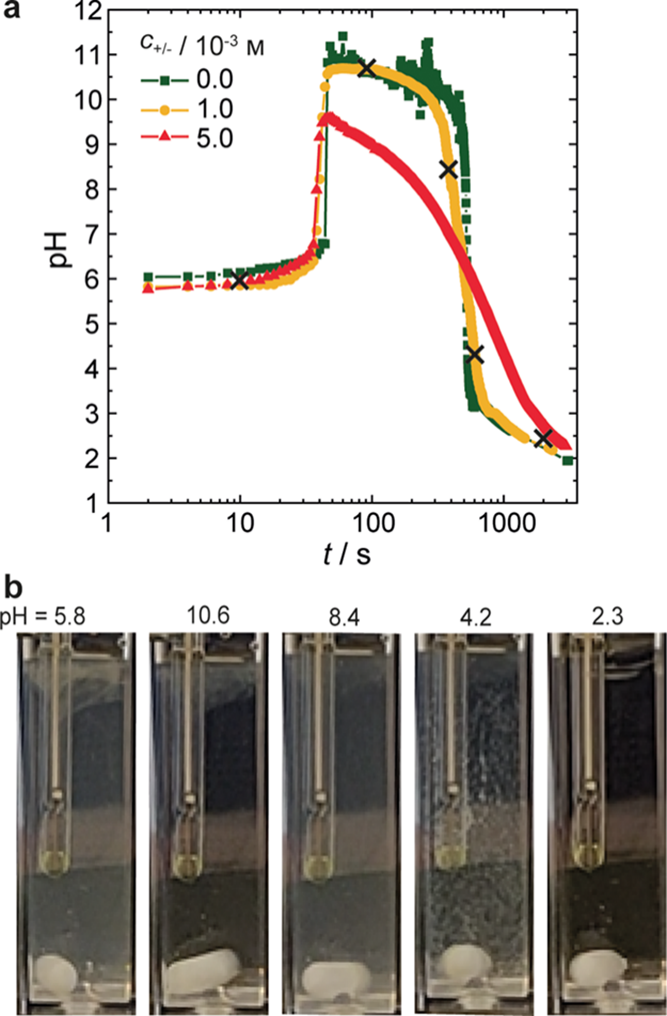
(a) temporal evolution of pH measured in samples containing up to 5 × 10^–3^m of PAH and PSM during the course of the F-S clock reaction and hydrolysis of PrS (0.2 m) on the logarithmic time scale (for the linear time scale, see Figure S4); (b) frames taken from a video at *t* = 10 s (pH 5.8), 90 s (pH 10.6), 360 s (pH 8.4), 600 s (pH 4.2), and 2100 s (pH 2.3) corresponding to the black crosses in panel a), for the sample with *c*_+/–_ = 10^–3^m.

Having tuned the F-S clock reaction to produce stable colloidal dispersions, we studied whether the lifetime of the nanoparticles could be programed by the addition of a transient acid-production reaction. To this end, we included 1,3-propanesultone (PrS) in the samples, just before formaldehyde addition. This cyclic sulfonic ester slowly hydrolyzes to a strong acid at the end of the F-S clock reaction (basic pH) and gradually lowers the pH. In the absence of the polymer, the clock reaction runs its predicted course, reaching a pH just above 10.5 after 48 s, followed by a plateau at high pH for a few minutes. Then, the hydrolysis of PrS (0.2 m) takes over and the acid production decreases the pH to well below 3 over the course of 14 min, with the steepest slope around 8 min (Figure 6a). Using both PAH and PSM at a concentration of *c*_+/–_ = 10^–3^m, there is little difference in the temporal evolution of the pH, apart from a more gradual slope in the downward section. However, the decrease in pH occurs more gradually, taking about twice as long (29 min) at a polyelectrolyte concentration of 5 × 10^–3^m.

Visually, the sample changes over the course of the reaction. Because of similar starting conditions, initial turbidity is again observed in the samples with PrS added (Figure 6b). This turbidity suddenly decreases as the pH shoots up to pH = 10.6. The subsequent decrease in pH, resulting from PrS hydrolysis, goes hand in hand with an increase in turbidity until a pH of 5–6, after which visible aggregates begin to appear in the solution that grow into flakes. In the absence of stirring, these flakes sediment. Upon stirring during acidification, the flakes redissolve and a clear solution remains at the end, at a pH around 2.3. These findings indicate that the highly stable nanoparticles obtained at high pH can be disassembled under very acidic conditions. The stability that partially originates from an excess negative charge is compromised as the pH is lowered by PrS. At sufficiently low PSM charge densities (at pH < 6 < pK_a,PSM_), aggregation of the particles is no longer prevented and large flakes form. A further decrease in pH weakens the supramolecular bonds within the complexes to such an extent that they ultimately disintegrate. The flakes then redissolve, and the system becomes transparent. This could indicate that in the present case, formaldehyde–PAH cross-linking is less pronounced or even absent.

**Figure 7:**
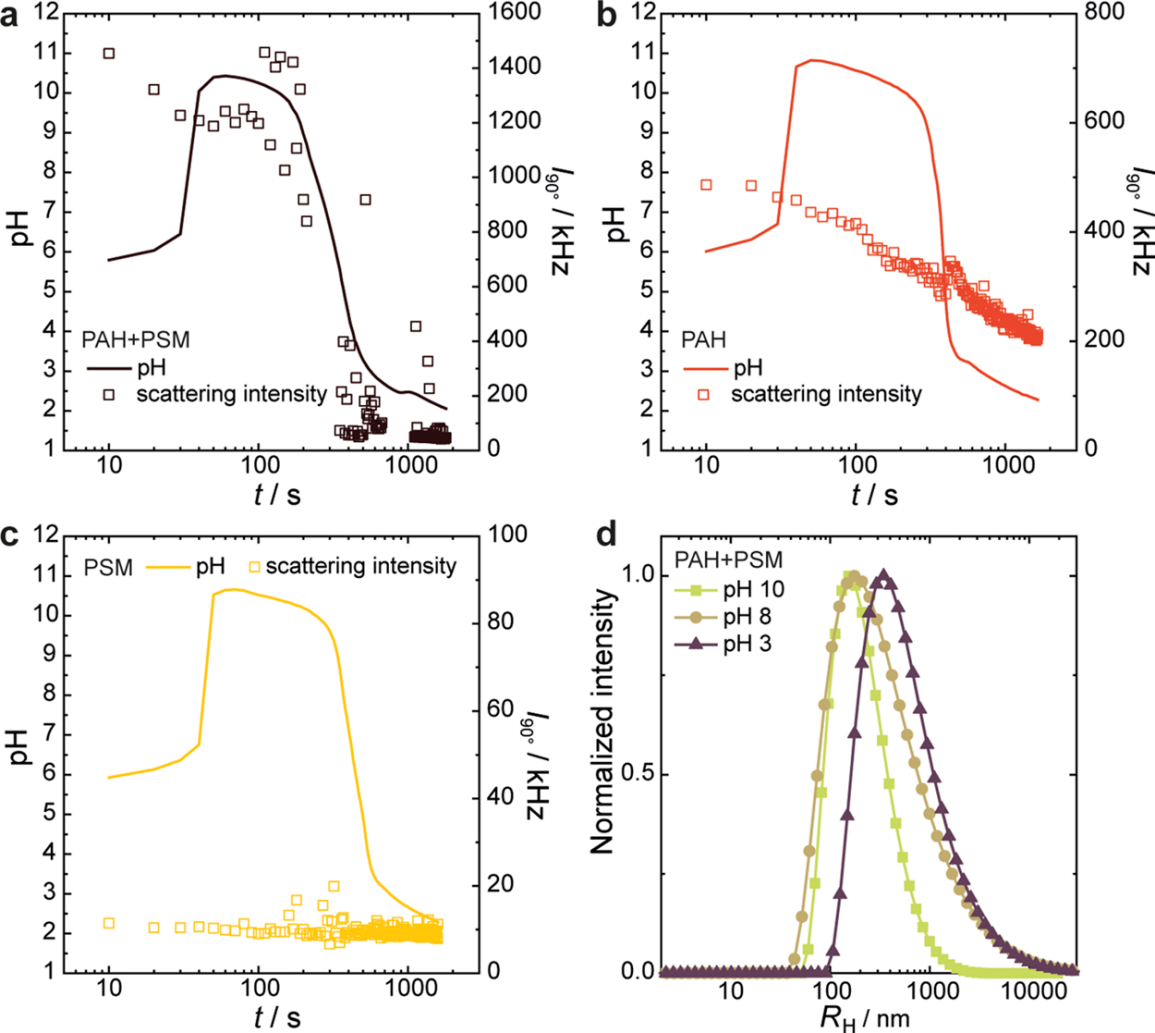
Light scattering intensity (symbols) and pH (lines) measured during the F-S clock reaction with PrS (0.2 m) hydrolysis of samples containing 10^–3^m of (a) both PAH and PSM, (b) only PAH, and (c) only PSM; (d) intensity-weighted size distributions determined for the sample containing both polyelectrolytes (panel a) at the time points where the pH was 10, 8, and 3, respectively.

To monitor whether the polyelectrolyte complexes fully dissolve as unimers through acidification and examine whether or not formaldehyde–PAH cross-linking occurs, we simultaneously recorded the static light scattering intensity and pH of samples, containing either PAH, PSM, or equal amounts of both polyelectrolytes (Figure 7). A pronounced scattering intensity is observed from the start in samples containing a mixture of both polyelectrolytes at *c*_+/–_ = 10^–3^ M. Scattering decreases slightly when the pH goes up, but quickly recovers (Figure 7a). Large aggregates start to form as the pH goes down, marked by a decrease of the scattering intensity, indicating fast sedimentation. The stirring in the sample cell does not prevent the large aggregates from quickly sedimenting, but some peaks in intensity are still observed when aggregates pass the laser beam. Full dissolution of the complexes is signaled by a low scattering intensity at pH < 3 (∼500 s) and the absence of sediment. At this point, the recorded scattering intensity is comparable to that of the solvent (i.e., in the absence of polymers). This result further confirms that formaldehyde-PAH cross-linking does not occur in PAH/PSM coacervates as in samples containing only PAH. In such complexes, the reactive amines are shielded by their interaction with the oppositely charged PSM units.

As expected, in the PAH solutions without PSM (Figure 7b), complexes of PAH and sulfite anions form as detected by its noticeable scattered light. These complexes are cross-linked by reactions with formaldehyde, as reported previously.^19^ Acidification leads to a decrease in scattering intensity, albeit less steep than for the PSM/PAH particles. After reaching pH = 2.2, many PAH particles remain, as the scattering intensity is still above the solvent level. In neat PSM solutions, the scattering intensity remains at the background level during the course of the clock reaction (Figure 7c). This is in accordance with expectations because PSM is soluble in the entire pH range and not prone to complexation with any of the constituents of the F-S clock. DLS revealed that the change in static light scattering intensity observed in the PAH/PSM mixtures as the pH starts to decrease due to PrS hydrolysis is accompanied by a shift in the particle size distributions (Figure 7d, correlation functions are plotted in Figure S5). Given the low scattering intensity measured at pH 3, the larger particles with a hydrodynamic radii of approximately 400 nm found at these acidic conditions are not abundant. Presumably, many of the polymer complexes have already dissolved at this point. A further decrease of pH to values close to 2 reduces the scattering signal to such low values that it is no longer possible to determine a correlation function of sufficient quality for particle sizing. These findings support the proposed mechanism of stabilization: above pH ∼ 8, electrostatic repulsion due to excess negative charge prevents aggregation, which is negated when acidification lowers the degree of ionization of PSM. The complete dissolution of the polymer complexes upon acidification to pH 2 suggests that formaldehyde cross-links might not occur as PAH amines are strongly interacting with PSM.

## Conclusions

To summarize, the assembly of polyelectrolyte nanostructures with tunable dimensions presents a challenge since the coacervation process often leads to macroscopic phase separation. Usually, restricting this phase separation to the colloidal domain is achieved by the covalent attachment of neutral polymer chains. We have demonstrated how PAH/PSM complexes can instead be restricted to the nanoscopic sizes by making use of the F-S clock reaction. This is achieved by kinetically trapping the nanometric complex coacervates formed upon mixing of the polyelectrolytes, PAH and PSM, at charge stoichiometry. The pH change induced by the clock reaction offsets the balance of charges but the coacervates do not dissociate at high pH. Rather, nanoparticles are produced with high colloidal stability. Complementary light scattering and transmission electron microscopy revealed that these nanoparticles have a hydrodynamic diameter of ca. 200 nm and are composed of a core–shell structure. In contrast, particles derived from the same polymers yet prepared through manual pH shift does not produce stable systems, demonstrating the essential role of the F-S clock to ensure long-term stability. Futhermore, we showed that the coacervates can be assembled transiently, by combining the F-S clock with the hydrolysis of 1,3-propanesultone. PrS hydrolysis results in a pH decrease following the initial increase by the clock reaction. The coacervates, that form in the (bi)sulfite buffer, are first transformed into nanoparticles by the F-S clock and subsequently destabilized as the PrS-induced acidification reduces the repulsive interactions. Consequently, the lack of stabilization leads to aggregate formation, while a further reduction of the pH leads to disassembly as the PSM becomes fully protonated so that the solution becomes transparent. This behavior strongly indicates that formaldehyde/PAH cross-linking is inhibited by the electrostatic interaction with PSM. This study shows that programming of the assembly pathway of complex coacervates through pH-regulating networks offers a versatile tool to create reversibly assembled nanoparticles with controlled dimensions, superb colloidal stability, and a tunable lifetime.

## Supporting Information Available

Degrees of ionization (α) as a function of pH for poly(sodium methacrylate), PSM, and poly(allylamine hydrochloride (PAH); area-normalized size distributions of PSM/PAH polyelectrolyte complexes; intensity-weighted size distributions; temporal evolution of pH measured in samples; and intensity autocorrelation functions of samples with PSM/PAH complexes (PDF)
