# Supplementary material for: Controlling the Formation of Polyelectrolyte Complex Nanoparticles Using Programmable pH Reactions
Source: Macromolecules. 2022 Dec 16;56(1):226–33. doi: 10.1021/acs.macromol.2c01431 (PMC9835975; doi:10.1021/acs.macromol.2c01431)
Supplement: Supplementary file 1 — ma2c01431_si_001.pdf [file ma2c01431_si_001.pdf]

Supporting information to:

Controlling the formation of polyelectrolyte complex nanoparticles using programmable pH-reactions.

*Christian C. M. Sproncken, Berta Gumí-Audenis, Sanam Foroutanparsa, José Rodrigo Magana, Ilja K. Voets**

Laboratory of Self-Organizing Soft Matter, Department of Chemical Engineering and Chemistry, and Institute for Complex Molecular Systems, Eindhoven University of Technology, PO Box 513, 5600 MB, Eindhoven, The Netherlands

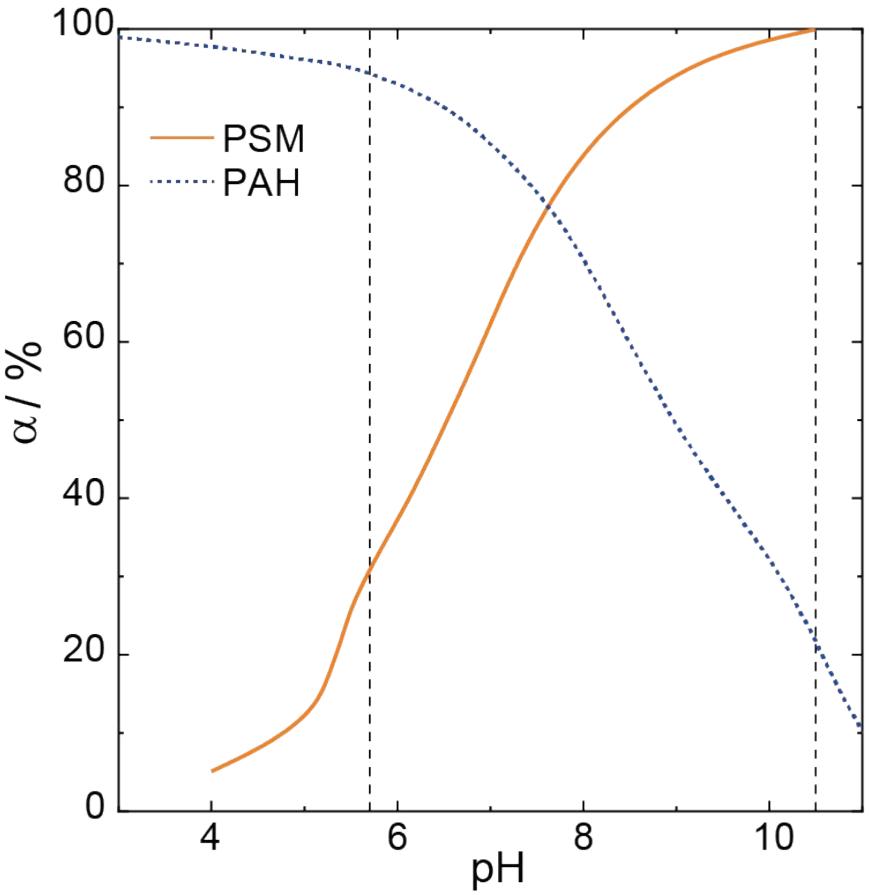

Figure S1. The degrees of ionization (α) as a function of pH for poly(sodium methacrylate), PSM, and poly(allylamine hydrochloride (PAH), based on data from Pohlmeier *et al*.[1] and Choi *et al*.[2] Dashed lines indicate starting and end pH (5.7 and 10.5) of the F-S clock reaction.

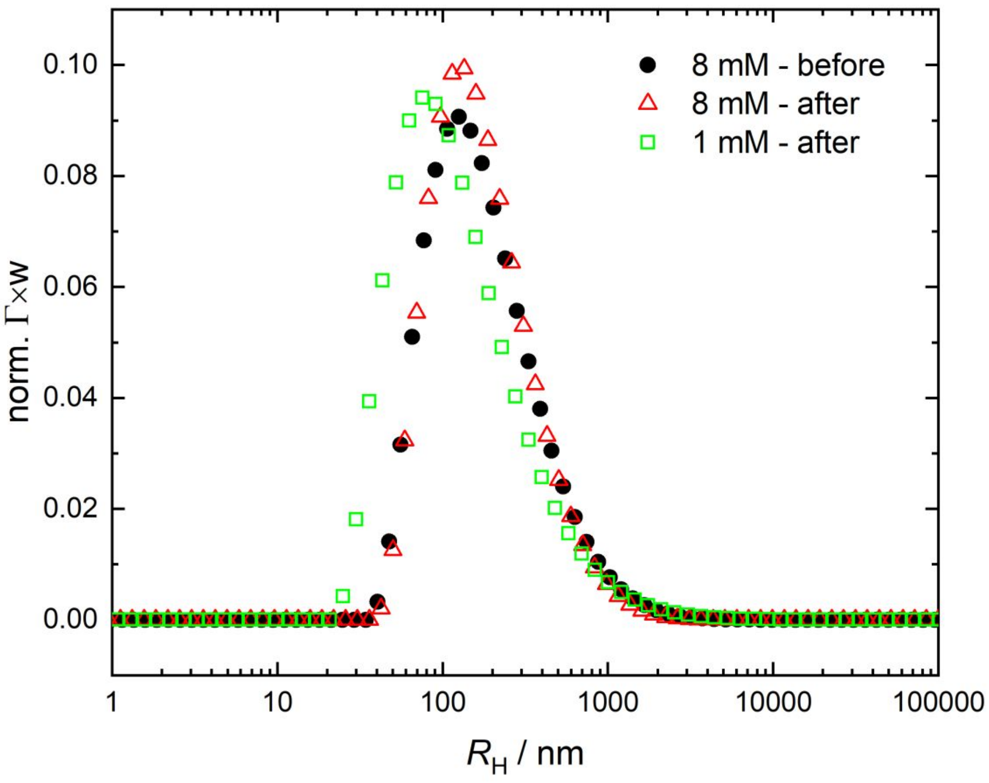

Figure S2. Area-normalized size distributions of PSM/PAH polyelectrolyte complexes at $8{\cdot}10^{-3}$ M, before and after the clock reaction, compared to $1{\cdot}10^{-3}$ M (after). The hydrodynamic sizes for the complexes at increased polymer concentration are a bit higher, yielding R_H = 140-150 nm, compared to 110 nm.

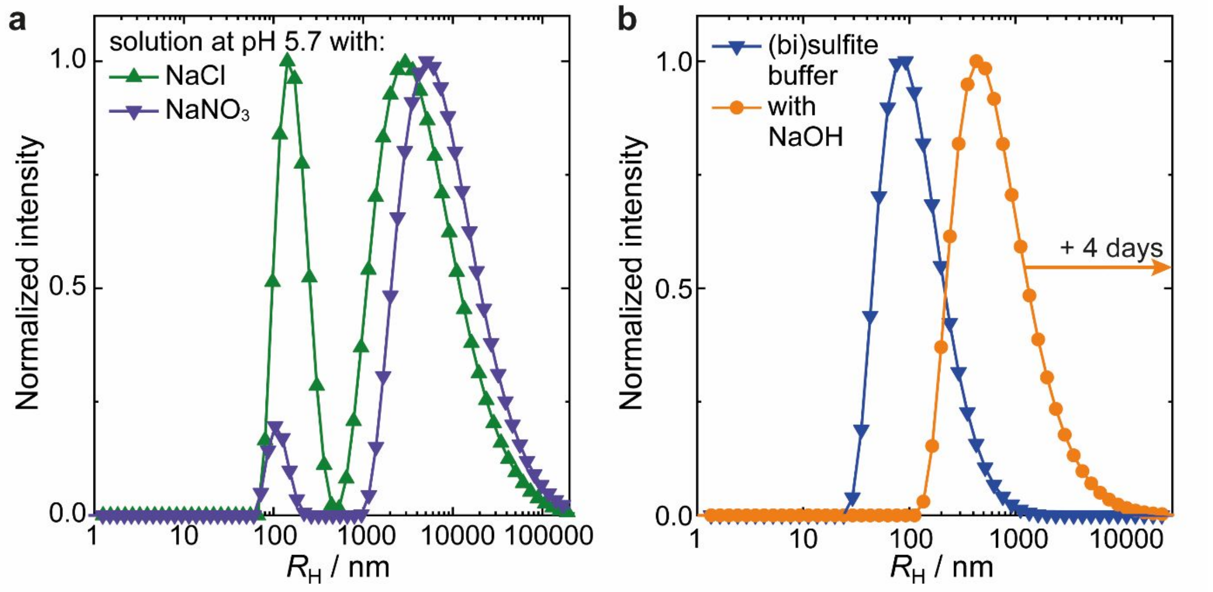

Figure S3. Intensity-weighted size distributions of PSM/PAH polyelectrolyte complexes after storage **a)** for 24 hours at pH 5.7 in 0.06 M sodium chloride (▲) or sodium nitrate (▼) and **b)** in sulfite-bisulfite buffer (0.005/0.05 M) before and after addition of NaOH solution to increase the pH from 5.7 (▼) to 10.5 (●). All these storage

conditions lead to significant growth and aggregation of the complexes. Storing the sample at pH 10.5 for 4 more days results in large complexes visible by eye.

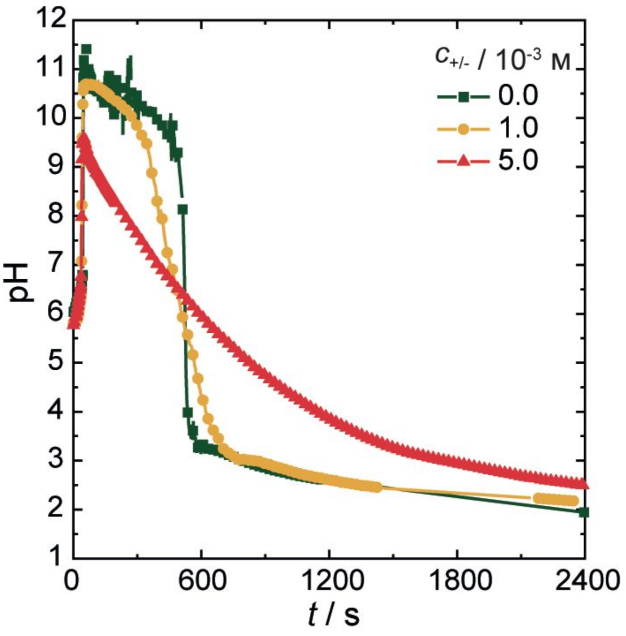

Figure S4. Temporal evolution of pH measured in samples containing up to $5 \cdot 10^{-3}$ M of PAH and PSM during the course of the F-S clock reaction and hydrolysis of PrS (0.2 M) on a linear time scale. Logarithmic time scale plot is found in the main text, **Figure 6**.

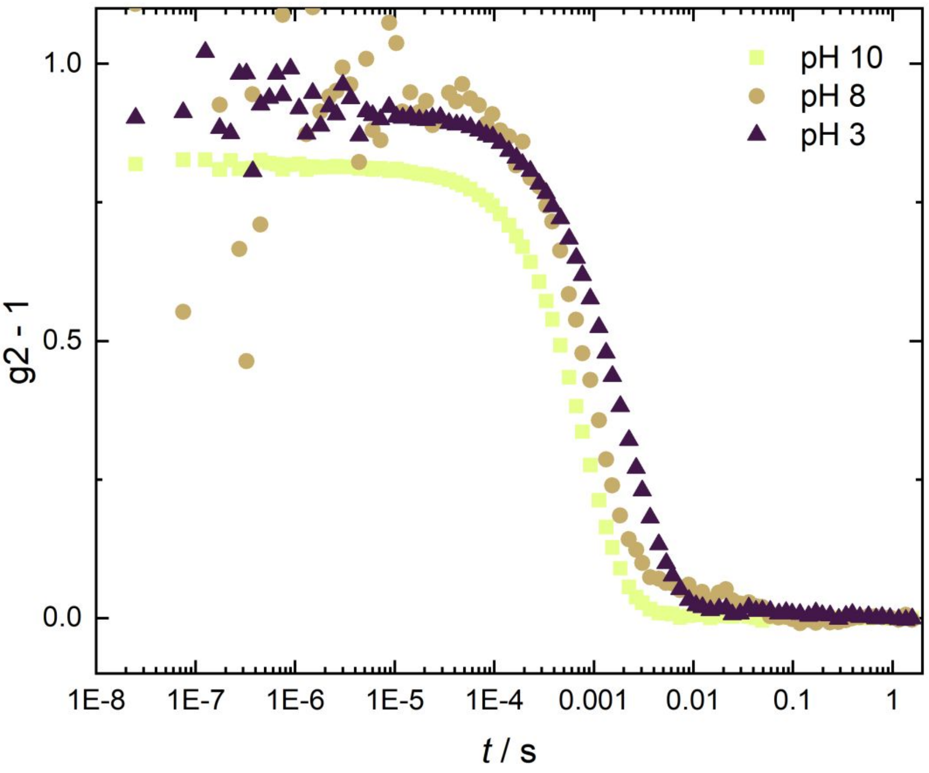

Figure S5. Intensity autocorrelation functions of samples with PSM/PAH complexes after the clock reaction, during slow acidification by PrS from pH = 10 to pH = 3. The shift of the decay to higher lag times shows increase in size and the bumps appearing during acidification, most clearly at pH 8, indicate the formation of aggregates.

References

(1) Pohlmeier, A.; Haber-Pohlmeier, S. Ionization of Short Polymethacrylic Acid: Titration, DLS, and Model Calculations. *J. Colloid Interface Sci.* **2004**, *273* (2), 369–380. https://doi.org/10.1016/j.jcis.2004.02.047.

(2) Choi, J.; Rubner, M. F. Influence of the Degree of Ionization on Weak Polyelectrolyte Multilayer Assembly. **2005**. https://doi.org/10.1021/ma048596o.
